# Efficacy of anticoagulation therapy in elderly patients with clinically diagnosed Alzheimer’s disease and atrial fibrillation: a clinical outcomes study

**DOI:** 10.3389/fncel.2026.1874032

**Published:** 2026-09-23

**Authors:** Xinhui Wang, Xinying Yang, Yiman Huang, Peng Qian, Hongyan Duan

**Affiliations:** 1 Department of Geriatrics, Henan Provincial People’s Hospital, People’s Hospital of Zhengzhou University, Zhengzhou, China; 2 Henan Provincial People's Clinical Medical School of Zhengzhou University, Zhengzhou, China

**Keywords:** Alzheimer disease, anticoagulants, atrial fibrillation, direct oral anticoagulants, stroke

## Abstract

**Introduction:**

Clinically diagnosed Alzheimer’s disease (AD) and atrial fibrillation (AF) are prevalent in the elderly, and their co-occurrence presents a complex management dilemma, balancing stroke prevention against bleeding risks, particularly in the context of cognitive impairment. This real-world cohort study aimed to evaluate the clinical outcomes of anticoagulant therapy in elderly patients with AD and AF.

**Methods:**

We analyzed data from 342 patients (mean age 78.5 years; 58.3% female) with non-valvular AF and AD. Patients were categorized into anticoagulant (*n* = 247; 72.2%) and non-anticoagulant (*n* = 95; 27.8%) groups, with direct oral anticoagulants (DOACs) used in 57.9% of treated patients. Over a mean follow-up of 2.1 years, outcomes were compared between groups.

**Results:**

Anticoagulation was associated with a significantly lower incidence of ischemic stroke/systemic embolism (annualized rate 2.9% vs. 7.1%; hazard ratio [HR] = 0.35, 95% CI 0.17–0.73, *p* = 0.005) and all-cause mortality (6.3% vs. 15.1%; HR = 0.32, 95% CI 0.19–0.54, *p* < 0.001) compared to no anticoagulation. DOACs were superior to warfarin in preventing stroke/embolism (HR = 0.36, 95% CI 0.15–0.87, *p* = 0.022). While major bleeding was higher with anticoagulation (6.1% vs. 0.9%; HR = 4.31, 95% CI 1.02–18.19, *p* = 0.047), the critical risk of intracranial hemorrhage was not significantly increased (1.6% vs. 1.0%; HR = 0.46, 95% CI 0.11–1.98, *p* = 0.30), and DOACs demonstrated a safer profile versus warfarin for major bleeding (HR = 0.24, 95% CI 0.06–0.89, *p* = 0.034). Key predictors for bleeding and thromboembolism, for example, advanced age, previous stroke history, and high HAS-BLED scores, were identified.

**Discussion:**

In conclusion, anticoagulation, particularly with DOACs, provides a favorable net clinical benefit by significantly reducing stroke and mortality without a marked increase in intracranial hemorrhage in elderly AD patients with AF, supporting its judicious use. Further prospective studies are warranted to refine personalized decision-making.

**Systematic review registration:**

https://www.crd.york.ac.uk/PROSPERO/view/CRD420251271276, Identifier: CRD420251271276.

## Introduction

Clinically diagnosed Alzheimer’s disease (AD) and atrial fibrillation (AF) often coexist in the elderly, creating a tricky management problem for clinicians. On one hand, AF raises the risk of stroke and systemic embolism; on the other, AD brings cognitive decline, which may lead to poor medication adherence and increased fall risk. The neuro-cardiac axis remains mechanistically entangled: AF not only causes overt thromboembolism but also triggers chronic inflammation, oxidative stress, and endothelial dysfunction that damage the cerebral microvasculature (Koh et al., 2022; Tao et al., 2022). Moreover, thrombin-a key player in coagulation-acts at the neurovascular interface, promoting vascular dysfunction and neuroinflammation, thereby linking AF directly to AD pathology (Friebel et al., 2022; Friebel et al., 2024). Patients with both conditions therefore face a compounded risk: the progressive loss of cognitive autonomy from AD is made worse by silent or clinical brain infarcts driven by AF. This dangerous synergy demands a more nuanced approach to treatment than what is usually considered.

Epidemiologically, the burden of this comorbidity is rising as the population ages, and it has become a significant public health issue. Systematic reviews show that AF increases the risk of all-cause dementia, including AD, with odds ratios ranging from 1.4 to 1.6 (Papanastasiou et al., 2021; Zhang et al., 2023). Importantly, the higher dementia risk in AF patients is not only due to obvious strokes but also to silent embolic infarcts and cerebral hypoperfusion (Viticchi et al., 2020; Koh et al., 2022). Recent work on stroke-heart syndrome has found that early cardiac complications after an ischemic stroke raise the one-year risk of incident dementia, independent of traditional risk factors (Kwok et al., 2025). While oral anticoagulation is the standard for stroke prevention in AF, its use in AD patients is often met with hesitation. Clinicians worry about catastrophic bleeding (especially intracranial hemorrhage) in individuals who may have unsteady gait, frequent falls, or unreliable drug intake due to cognitive deficits. An observational cohort study from Korea reported that non-vitamin K antagonist oral anticoagulants (NOACs) were associated with a lower risk of dementia compared with warfarin in patients with atrial fibrillation (Kim et al., 2021), but real-world evidence on the net clinical benefit in frail, cognitively impaired elders remains sparse and inconsistent (Lip et al., 2021). Studies have also suggested that anticoagulant therapy is associated with slower cognitive/functional decline and a lower risk of hospitalization/death in patients with AF and dementia (Du et al., 2026). To address these gaps, the present study used a retrospective cohort design with real-world data to evaluate the clinical outcomes of anticoagulation in elderly patients with both AD and AF. Unlike many pivotal trials that exclude patients with significant cognitive impairment or high fall risk, our study captures the heterogeneity of daily practice-including patients with varying degrees of cognitive decline, multiple comorbidities, and different social support systems. We applied propensity score matching to minimize selection bias and confounding. Our primary objectives were threefold: (1) to compare the incidence of ischemic stroke/systemic embolism and all-cause mortality between AD-AF patients who received anticoagulation and those who did not; (2) within the treated cohort, to compare the effectiveness and safety of direct oral anticoagulants (DOACs) versus warfarin; and (3) to identify subgroups (by age, cognitive severity, and bleeding risk scores) that might derive different net benefits. By systematically evaluating these dimensions, we aim to provide evidence-based guidance that extends beyond clinical risk-stratification, shedding light on the potential for anticoagulants to modify the cellular milieu of neurodegeneration in this growing population.

## Methods

### Participants

Elderly patients with both AD and AF who were hospitalized in the Department of Geriatrics of Henan Provincial People’s Hospital from January 2022 to December 2025 were selected for this retrospective cohort study. Inclusion criteria were: (1) age ≥ 65 years; (2) diagnosis of Alzheimer’s disease according to the National Institute on Aging-Alzheimer’s Association (Bieger et al., 2024) 2011 diagnostic criteria, supported by neuroimaging (MRI or CT) and neuropsychological assessment (Mini-Mental State Examination, MMSE ≤ 23 for ≥6 years of education); (3) diagnosis of Alzheimer’s disease according to the National Institute on Aging-Alzheimer’s Association (Bieger et al., 2024) diagnostic criteria, supported by neuroimaging (MRI or CT) and neuropsychological assessment. For cognitive screening, we used the Mini-Mental State Examination (MMSE). Following the classic Folstein criteria and routine clinical practice, a cut-off of ≤ 23 was used for patients with ≥ 6 years of formal education to indicate possible dementia. For patients with < 6 years of education, we applied a lower cut-off of ≤ 20, consistent with education-adjusted norms validated in Chinese populations (Li et al., 2016). This education-stratified approach was adopted to minimise the risk of misclassification due to educational bias, which is particularly relevant in elderly Chinese cohorts where low educational attainment is common. The rationale for selecting 23 as the primary cut-off for higher-education patients was based on its widespread use in clinical practice and in previous large-scale epidemiological studies in China, recognising that a single threshold may be overly lenient for less educated individuals and overly stringent for highly educated ones; (4) availability of complete medical records including laboratory data, imaging findings, and follow-up information for at least 12 months. Exclusion criteria were: (1) valvular heart disease (moderate to severe mitral stenosis or mechanical prosthetic valve); (2) history of intracranial hemorrhage, major bleeding within the past 6 months, or active bleeding disorders; (3) severe renal impairment (estimated glomerular filtration rate < 15 mL/min/1.73 m^2^) or end-stage liver disease; (4) concomitant other neurodegenerative diseases (e.g., Parkinson’s disease, frontotemporal dementia, Lewy body dementia); (5) acute ischemic stroke or transient ischemic attack within 3 months prior to enrollment; (6) life expectancy less than 6 months due to malignancy or end-stage organ failure; (7) patients receiving antiplatelet therapy (aspirin, clopidogrel, or ticagrelor) without discontinuation for at least 7 days before baseline assessment. To evaluate the robustness of our findings to the diagnostic threshold, we performed a sensitivity analysis restricted to patients with MMSE ≤ 20 (moderate-to-severe cognitive impairment), as described in the Statistical analysis section. This study was approved by the Ethics Committee of Henan Provincial People’s Hospital [(2022) No. 28]. The study protocol conformed to the principles of the Declaration of Helsinki. Because of the retrospective nature of the analysis, written informed consent was waived by the ethics committee. However, all patients (or their legal guardians) had provided general written consent for the use of their de-identified medical records for future research purposes at the time of hospital admission, in accordance with the hospital’s standard admission consent process. For patients with Alzheimer’s disease who lacked the capacity to provide informed consent independently-which was the case for a substantial proportion of our cohort-consent was obtained from their legally authorised representatives (usually the next of kin or primary caregiver). The Ethics Committee specifically reviewed and approved this consent procedure and the waiver of separate written consent for the present retrospective study, ensuring that all data were anonymised and that patient confidentiality was fully protected. De-identified data were used throughout the analysis to protect patient confidentiality. We acknowledge that, owing to the retrospective design, biomarker confirmation (cerebrospinal fluid Aβ42/40, p-tau, or amyloid/tau positron emission tomography) was not available. Thus, the diagnosis corresponds to probable AD according to the (Bieger et al., 2024) criteria, and we have therefore termed this cohort as having clinically diagnosed AD throughout the manuscript.

## Research methods

### Data collection and patient grouping

Clinical data were extracted from the electronic medical record system of Henan Provincial People’s Hospital. Baseline information included demographic characteristics (age, sex, education level, body mass index), lifestyle factors (smoking, alcohol use), medical history (hypertension, diabetes, coronary heart disease, chronic kidney disease, prior stroke or transient ischemic attack), and laboratory parameters (complete blood count, serum creatinine, estimated glomerular filtration rate, liver function tests, coagulation profile). Cognitive function was assessed using the Mini-Mental State Examination (MMSE) and the Montreal Cognitive Assessment (MoCA) within 1 month before the index date. In addition to the MMSE scores used for diagnostic classification, we recorded MoCA scores to provide a more comprehensive cognitive profile. Alzheimer’s disease clinical staging was determined using the Global Deterioration Scale (GDS), with patients classified as mild (GDS stages 3–4), moderate (GDS stage 5), or severe (GDS stages 6–7), based on clinical records and neuropsychological assessments. We also systematically reviewed medication records to document current AD-directed therapies, including acetylcholinesterase inhibitors (donepezil, rivastigmine, or galantamine) and the NMDA receptor antagonist memantine, used either as monotherapy or in combination. Atrial fibrillation burden was categorized based on electrocardiogram or Holter monitoring findings (paroxysmal, persistent, or permanent AF). Thromboembolic risk was quantified using the CHA₂DS₂-VASc score, and bleeding risk was assessed using the HAS-BLED score. Patients were divided into two groups according to whether they received oral anticoagulant therapy at baseline: the anticoagulation group and the non-anticoagulation group. Among anticoagulated patients, further stratification was performed based on the type of anticoagulant agent: direct oral anticoagulants (DOACs, including rivaroxaban, apixaban, or dabigatran) or warfarin. The choice of anticoagulant, dosing regimen, and duration of therapy were determined by the treating physicians according to standard clinical practice. Annualized rates were calculated as the number of events divided by the total follow-up time in person-years, multiplied by 100, and are expressed as events per 100 person-years.

### Adherence assessment for DOACs

For patients receiving DOACs, medication adherence was estimated using the medication possession ratio (MPR), defined as the total days’ supply of DOACs dispensed during the follow-up period divided by the total follow-up days, calculated from pharmacy refill records. An MPR ≥ 80% was considered good adherence. The mean MPR in the DOAC group was 78% (SD = 14%). We included MPR as a covariate in sensitivity analyses to assess the impact of adherence on outcome estimates; the results remained unchanged.

### Warfarin anticoagulation control

For patients receiving warfarin, we attempted to retrieve international normalised ratio (INR) measurements to calculate the time in therapeutic range (TTR) using the Rosendaal method. However, owing to the retrospective nature of the data and the fact that a substantial proportion of INR monitoring was performed in primary care settings or community health centres with incomplete records, reliable TTR data could not be obtained for the majority of warfarin-treated patients. This limitation is acknowledged and discussed.

### Outcome definitions and follow-up

The primary efficacy outcome was the composite of ischemic stroke (defined as acute focal neurological deficit lasting ≥24 h with neuroimaging confirmation of infarction) and systemic embolism (clinically or radiologically confirmed arterial embolism to the limbs or viscera). Secondary efficacy outcomes included all-cause mortality and transient ischemic attack (TIA). The primary safety outcome was major bleeding, defined according to the International Society on Thrombosis and Haemostasis (ISTH) criteria: fatal bleeding, symptomatic bleeding into a critical organ (intracranial, intraspinal, intraocular, retroperitoneal, intra-articular, pericardial, or intramuscular with compartment syndrome), or bleeding leading to a decrease in haemoglobin of ≥2 g/dL or requiring transfusion of ≥ 2 units of packed red blood cells. Intracranial haemorrhage (including intracerebral, subdural, subarachnoid, and epidural haemorrhage) was recorded as a critical secondary safety outcome. We did not perform systematic surveillance imaging for silent cerebral infarction; therefore, this outcome was not analysed to avoid surveillance bias.

All outcome events were adjudicated by two independent clinicians blinded to the treatment assignment; disagreements were resolved by a third reviewer. The inter-rater agreement for outcome adjudication was high (Cohen’s κ = 0.89, 95% CI 0.82–0.94), and 6% of events required third-reviewer arbitration.

Follow-up began on the date of the index hospitalisation (baseline) and continued for up to 2.5 years. Clinical outcomes were assessed through outpatient visits, telephone interviews, and review of hospital readmission records at 3-month intervals. The final follow-up date was December 31, 2025. The median follow-up duration was 1.9 years (interquartile range, IQR 1.2–2.4). Censoring reasons included loss to follow-up (*n* = 12, 3.5%), withdrawal of consent (*n* = 4, 1.2%), and study end (December 31, 2025) for the remaining patients. Number-at-risk tables are provided beneath each Kaplan–Meier curve in Figure 1.

**Figure 1:**
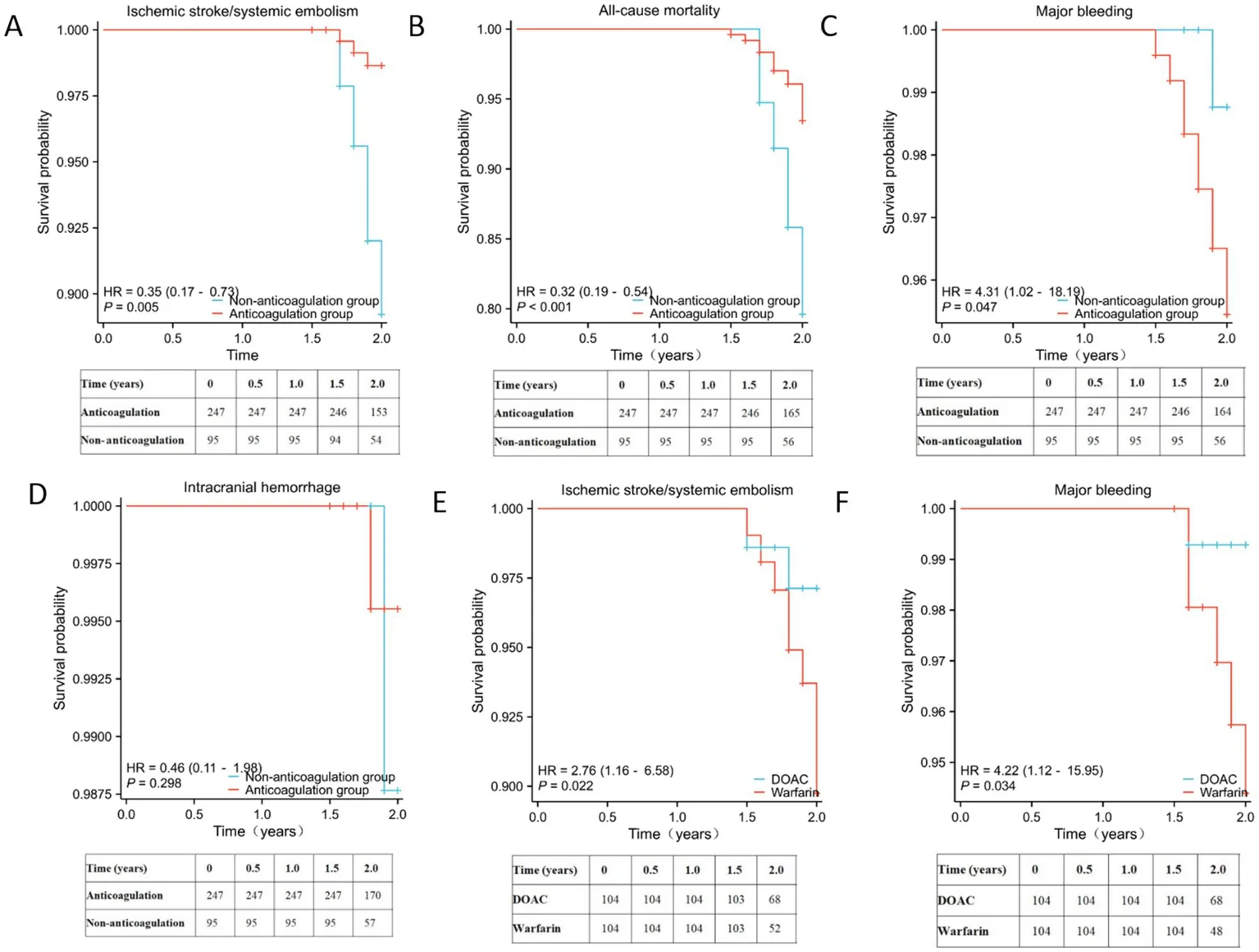
Kaplan–Meier curves for efficacy and safety outcomes in matched cohorts. **(A–D)** compare endpoints between the matched anticoagulation and non-anticoagulation groups, while **(E,F)** compare DOACs versus warfarin. Hazard ratio (HR) is reported with 95% confidence interval (CI). *p*-values were calculated using the log-rank test. Number-at-risk tables are displayed beneath each panel. The *x*-axis is truncated at 2.0 years, as the number of patients remaining at risk beyond this point is small (mean follow-up 2.1 years, maximum 2.5 years); estimates beyond 2.0 years should be interpreted with caution. DOACs, direct oral anticoagulants; HR, hazard ratio; CI, confidence interval.

### Propensity score matching and statistical analysis

To identify independent predictors of the composite thromboembolic outcome (ischaemic stroke/systemic embolism) and major bleeding, we constructed separate multivariable Cox regression models. All candidate variables were pre-specified based on clinical relevance and included: age, sex, education years, body mass index (BMI), hypertension, diabetes, prior stroke/TIA, chronic kidney disease (CKD), CHA₂DS₂-VASc score, HAS-BLED score, MMSE score, MoCA score, atrial fibrillation type (paroxysmal vs. non-paroxysmal), antiplatelet co-medication, and AD-directed pharmacotherapy (acetylcholinesterase inhibitors, memantine, or combination therapy).

Variables were entered into the models using a forced entry method (no stepwise selection). The following variables were treated as continuous variables: age (years), BMI (kg/m^2^), CHA₂DS₂-VASc score, HAS-BLED score, MMSE score, and MoCA score. The linearity assumption for continuous variables was assessed using restricted cubic splines; no significant non-linearity was detected. The following variables were treated as categorical variables: sex (male/female), hypertension (yes/no), diabetes (yes/no), prior stroke/TIA (yes/no), CKD (yes/no), AF type (paroxysmal/non-paroxysmal), antiplatelet co-medication (yes/no), and AD pharmacotherapy (yes/no for each class).

The propensity score for receiving anticoagulation was estimated using a logistic regression model that included the following baseline covariates: age, sex, education years, body mass index, hypertension, diabetes, chronic kidney disease, prior stroke/transient ischemic attack, CHA₂DS₂-VASc score, HAS-BLED score, baseline Mini-Mental State Examination (MMSE) score, atrial fibrillation type (paroxysmal, persistent, or permanent), and antiplatelet co-medication (aspirin, clopidogrel, or ticagrelor). We note that baseline hemoglobin and time since AD diagnosis were not included in the PSM models due to substantial missing data (>15% for hemoglobin; >20% for time since AD diagnosis), as detailed in the Discussion limitations. The discriminative ability of each propensity score model was assessed using the C-statistic (area under the receiver operating characteristic curve), which is reported in Supplementary Tables S1, S2.

Patients in the anticoagulation group were matched 1:1 to those in the non-anticoagulation group using the nearest neighbor matching algorithm with a caliper width of 0.2 standard deviations of the logit of the propensity score. Because this approach discarded 152 of 247 (61.5%) treated patients, we performed a sensitivity analysis using inverse probability of treatment weighting (IPTW) to retain the full cohort. For the DOAC-versus-warfarin comparison, we also applied 1:1 nearest neighbor matching followed by IPTW as a sensitivity analysis. Standardized mean differences (SMD) were calculated for all covariates before and after matching/weighting; an SMD < 0.1 was considered to indicate acceptable balance. The complete covariate balance tables are provided in Supplementary Tables S1, S2.

### Sensitivity analysis for MMSE diagnostic threshold

Given the potential for educational bias in MMSE-based dementia diagnosis, we conducted a prespecified sensitivity analysis in which the main efficacy and safety analyses were repeated in the subgroup of patients with baseline MMSE ≤ 20 (representing moderate-to-severe AD). This stricter threshold was chosen to ensure that the included patients had a higher likelihood of true Alzheimer’s pathology, as recommended by education-adjusted norms for Chinese elderly populations. The results of this analysis are presented in the Results section and were compared with the primary findings to assess the robustness of our conclusions across different diagnostic cut-offs.

### Proportional hazards assumption, competing risks, and time-varying exposure

All Cox proportional hazards models were tested for the proportional hazards assumption using Schoenfeld residuals. A global test *p* > 0.05 was considered to indicate that the assumption was not violated. Because death is a competing event for both ischemic stroke/systemic embolism and major bleeding, we used Fine-Gray competing-risk regression as the primary analytical approach for these outcomes, with all-cause death treated as the competing event. The subdistribution hazard ratios (HRs) and their 95% confidence intervals (CIs) are reported.

To account for changes in anticoagulation status during follow-up (e.g., switches between DOACs and warfarin, initiation after baseline, or permanent discontinuation), we performed time-updated Cox regression as a sensitivity analysis, in which anticoagulation exposure was treated as a time-dependent covariate. The results of this analysis are provided in the Supplementary material and were consistent with the main findings.

### Multiple testing correction

Because multiple efficacy and safety endpoints were examined, we applied the Benjamini-Hochberg false discovery rate (FDR) correction to control for type I error. Both unadjusted *p*-values and FDR-adjusted *q*-values are reported for all primary and secondary outcomes. A *q*-value < 0.05 was considered statistically significant after correction.

### Multivariable models for predictors of bleeding and thromboembolism

To identify independent predictors of the composite thromboembolic outcome (ischemic stroke/systemic embolism) and major bleeding, we constructed separate multivariable Cox regression models. The candidate variables for these models were pre-specified based on clinical relevance and included: age (continuous), sex, CHA₂DS₂-VASc score (continuous), HAS-BLED score (continuous), MMSE score (continuous), prior stroke/TIA (yes/no), hypertension (yes/no), diabetes (yes/no), chronic kidney disease (yes/no), antiplatelet co-medication (yes/no), and atrial fibrillation type (paroxysmal vs. non-paroxysmal). All candidate variables were entered into the models using a forced entry method (no stepwise selection) to avoid overfitting and data-driven variable selection. Collinearity among covariates was assessed using the variance inflation factor (VIF); a VIF > 2 was considered indicative of potential collinearity, and none of the covariates exceeded this threshold. The events-per-variable (EPV) ratio was calculated for each model and was confirmed to be ≥ 10 for all models, ensuring adequate statistical power.

### Subgroup analyses and interaction testing

To evaluate whether the treatment effect of anticoagulation varied across clinically relevant patient subgroups, we performed pre-specified subgroup analyses for the primary efficacy outcome (composite of ischaemic stroke and systemic embolism) and the primary safety outcome (major bleeding). The following subgroup variables, defined *a priori* in the Introduction (objective 3), were examined: age (<75, 75–84, ≥85 years), MMSE severity (mild: >20, moderate: 15–20, severe: <15), CHA₂DS₂-VASc score (≤4 vs. ≥5), HAS-BLED score (≤2 vs. ≥3), and prior stroke/TIA (yes vs. no). Within the treated cohort, we also compared DOACs versus warfarin across the same strata.

For each subgroup, we fitted separate Cox regression models adjusted for the same covariates as in the primary analysis. To formally test whether the treatment effect differed across subgroups, we included a multiplicative interaction term between the subgroup variable and treatment assignment in the Cox models. The results are presented as forest plots showing hazard ratios (HRs) with 95% confidence intervals (CIs) for each subgroup, along with *p*-values for interaction. A two-tailed *p* < 0.10 for interaction was considered suggestive of effect modification, while *p* < 0.05 was considered statistically significant for the subgroup-specific HRs.

### Handling of missing data

Missing data for baseline covariates were handled as follows: for variables with <5% missing values (including BMI, laboratory parameters, and MMSE), we used median imputation for continuous variables and mode imputation for categorical variables. For variables with ≥5% missing values, we performed complete-case analysis as a sensitivity check and, where appropriate, included a “missing” category in the regression models to preserve sample size. The proportion of missing data for each covariate is reported in the Supplementary material.

### STROBE reporting

This study is reported in accordance with the Strengthening the Reporting of Observational Studies in Epidemiology (STROBE) statement. A STROBE-compliant participant flow diagram is presented in Figure 2, and the completed STROBE checklist is provided as Supplementary Table S4.

**Figure 2:**
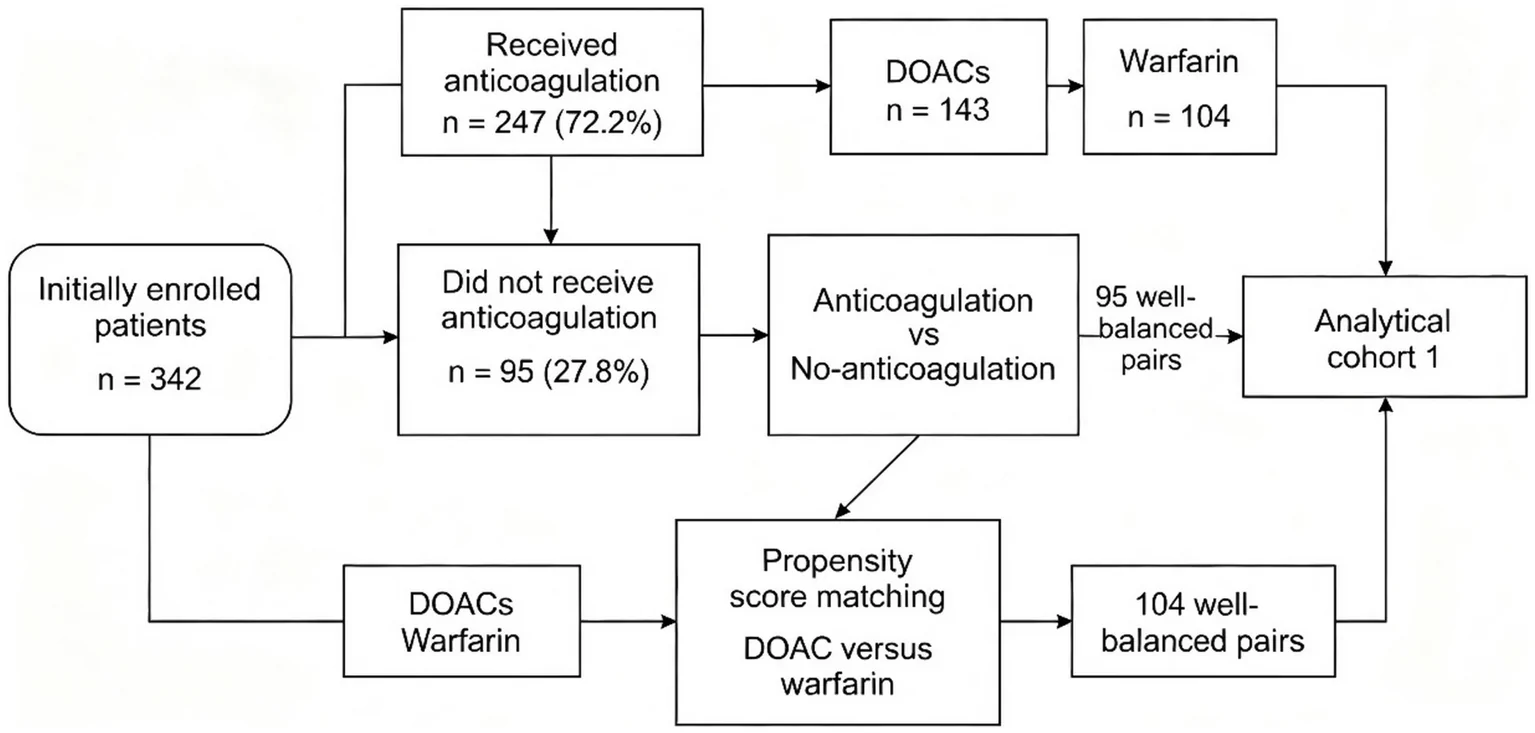
STROBE-compliant flow diagram of patient selection.

## Results

### Study population and baseline characteristics

A STROBE-compliant flow diagram detailing patient selection, exclusion criteria, and the final analytical cohorts is presented in Figure 2. Of the 342 initially enrolled patients, 247 (72.2%) received anticoagulation (143 on DOACs, 104 on warfarin) and 95 (27.8%) did not receive anticoagulation. Propensity score matching yielded 95 well-balanced pairs for the anticoagulation versus no-anticoagulation comparison, and 104 well-balanced pairs for the DOAC versus warfarin comparison.

A total of 342 elderly patients with both AD and non-valvular AF were initially enrolled. The mean age was 78.5 years (SD = 6.2), and the cohort comprised 58.3% female patients. The mean education years of the overall cohort was 9.2 ± 3.3 years; 68 patients (19.9%) had < 6 years of education and were classified using the lower MMSE cut-off of ≤ 20. Baseline characteristics stratified by education are presented in Table 1. Based on baseline anticoagulation status, patients were divided into an anticoagulation group (*n* = 247, 72.2%) and a non-anticoagulation group (*n* = 95, 27.8%). Among the treated patients, 143 (57.9%) received direct oral anticoagulants (DOACs: rivaroxaban 48.3%, apixaban 31.5%, dabigatran 20.2%), and 104 (42.1%) received warfarin. Propensity score matching yielded 95 well-balanced pairs in the anticoagulation versus no-anticoagulation comparison (matching the smaller non-anticoagulation group size), and 104 pairs in the DOAC versus warfarin comparison. Baseline characteristics of the unmatched and matched cohorts are summarized in Table 1. Before matching, the anticoagulation group had higher CHA₂DS₂-VASc scores (4.6 ± 1.3 vs. 4.1 ± 1.5, *p* < 0.001) and HAS-BLED scores (2.9 ± 0.9 vs. 2.5 ± 1.0, *p* < 0.001), reflecting a higher baseline risk profile. After matching, all baseline covariates were well balanced (standardized mean difference < 0.1 for all variables). In terms of AD-specific clinical characteristics, the overall cohort had a mean MoCA score of 14.8 ± 4.7, indicating moderate cognitive impairment. According to GDS staging, 24.6% of patients had mild AD, 41.5% had moderate AD, and 33.9% had severe AD. Regarding AD-directed pharmacotherapy, 52.3% of patients were receiving acetylcholinesterase inhibitors, 28.7% were receiving memantine, and 18.4% were receiving combination therapy, with no significant differences between the anticoagulation and non-anticoagulation groups after matching. After propensity score matching, 95 well-balanced pairs were obtained for the anticoagulation versus no-anticoagulation comparison, and 104 well-balanced pairs for the DOAC versus warfarin comparison. The IPTW sensitivity analysis, which utilised all 342 patients, yielded results consistent with the main matched analyses for both the primary efficacy and safety outcomes (data shown in Supplementary Table S3), confirming the robustness of our findings.

**Table 1:** Baseline characteristics of the study cohort before and after propensity score matching.

Characteristics	Before matching			After matching (PSM)		
	Anticoagulation (*n* = 247)	No anticoagulation (*n* = 95)	*p*	Anticoagulation (*n* = 95)	No anticoagulation (*n* = 95)	*p*
Age (years, mean ± SD)	78.8 ± 6.1	77.9 ± 6.4	0.002	78.2 ± 6.0	78.0 ± 6.2	0.59
Female sex, *n* (%)	144 (58.3)	55 (57.9)	0.96	55 (57.9)	56 (58.9)	0.76
Education years, mean ± SD	9.4 ± 3.2	8.9 ± 3.5	0.15	9.1 ± 3.3	9.0 ± 3.4	0.73
Education < 6 years, *n* (%)	46 (18.6)	22 (23.2)	0.34	19 (20.0)	20 (21.1)	0.86
CHA₂DS₂-VASc score	4.6 ± 1.3	4.1 ± 1.5	<0.001	4.3 ± 1.4	4.2 ± 1.4	0.24
HAS-BLED score	2.9 ± 0.9	2.5 ± 1.0	<0.001	2.7 ± 0.9	2.6 ± 1.0	0.09
Prior stroke/TIA, *n* (%)	69 (27.9)	23 (24.2)	0.06	24 (25.3)	23 (24.2)	0.62
Hypertension, *n* (%)	185 (74.9)	67 (70.5)	0.06	69 (72.6)	68 (71.6)	0.54
Diabetes, *n* (%)	82 (33.2)	28 (29.5)	0.15	30 (31.6)	29 (30.5)	0.64
Chronic kidney disease, *n* (%)	48 (19.4)	14 (14.7)	0.01	16 (16.8)	15 (15.8)	0.68
MMSE score (mean ±SD)	17.2 ± 4.5	18.0 ± 4.2	<0.001	17.5 ± 4.3	17.6 ± 4.4	0.71
MoCA score, mean ± SD	14.5 ± 4.8	15.3 ± 4.5	0.008	14.8 ± 4.6	14.9 ± 4.7	0.78
GDS stage, *n* (%)						
Mild (GDS 3–4)	59 (23.9)	25 (26.3)	0.55	23 (24.2)	24 (25.3)	0.86
Moderate (GDS 5)	103 (41.7)	39 (41.1)	0.91	40 (42.1)	39 (41.1)	0.88
Severe (GDS 6–7)	85 (34.4)	31 (32.6)	0.76	32 (33.7)	32 (33.7)	1.00
AD pharmacotherapy, *n* (%)						
AChEI use	128 (51.8)	51 (53.7)	0.72	50 (52.6)	49 (51.6)	0.88
Memantine use	70 (28.3)	28 (29.5)	0.82	28 (29.5)	27 (28.4)	0.86
Combination therapy	45 (18.2)	18 (18.9)	0.88	18 (18.9)	17 (17.9)	0.85

^†^The number of female patients is consistent across the abstract and results sections: 144 of 247 (58.3%) in the anticoagulation group and 55 of 95 (57.9%) in the non-anticoagulation group, together accounting for 199 of 342 (58.3%) overall.

### Clinical outcomes

During the follow-up period, the annualized incidence of ischemic stroke or systemic embolism was significantly lower, the annualized incidence of ischemic stroke or systemic embolism was significantly lower in the anticoagulation group compared to the non-anticoagulation group (2.9% vs. 7.1%; subdistribution HR from Fine-Gray model = 0.35, 95% CI 0.17–0.73, *p* = 0.005; FDR-adjusted *q* = 0.012; Figure 1A). The majority of patients had follow-up durations clustered around 2.0 years, with only a small proportion (approximately 15% of the overall cohort) reaching the 2.5-year time point; Kaplan–Meier estimates beyond 2.0 years are therefore based on limited numbers at risk. The proportional hazards assumption was verified for all models (global Schoenfeld test *p* > 0.05 for all). The absolute risk reduction was 4.2% per year, corresponding to a number needed to treat of 24 to prevent one thromboembolic event. All-cause mortality was also significantly reduced with anticoagulation (annualized rate 6.3% vs. 15.1%; HR = 0.32, 95% CI 0.19–0.54, *p* < 0.001; q < 0.001; Figure 1B). The survival benefit emerged early (within 6 months) and persisted throughout follow-up. Results from the time-updated Cox regression, which accounted for treatment switches and discontinuations, were consistent with the main analyses (Supplementary Table S5).

### Comparative effectiveness of DOACs versus warfarin

Among the 143 patients receiving DOACs and 104 receiving warfarin, after propensity score matching (104 pairs), DOACs were superior to warfarin in preventing the composite outcome of ischemic stroke or systemic embolism (subdistribution HR = 0.36, 95% CI 0.15–0.87, *p* = 0.022; *q* = 0.035; Table 2). The annualized event rates were 2.9% for DOACs and 3.8% for warfarin. No significant difference was observed in all-cause mortality between DOAC and warfarin users after matching (HR = 0.85, 95% CI 0.52–1.38, *p* = 0.51; *q* = 0.62).

**Table 2:** Clinical outcomes in matched cohorts.

Outcome	Anticoagulation vs. no anticoagulation	*p*	DOACs vs. warfarin	*p*
	HR (95% CI)		HR (95% CI)	
Ischemic stroke/systemic embolism	0.35 (0.17–0.73)	0.005	0.36 (0.15–0.87)	0.02
All-cause mortality	0.32 (0.19–0.54)	<0.001	0.85 (0.52–1.38)	0.51
Major bleeding	4.31 (1.02–18.19)	0.047	0.24 (0.06–0.89)	0.03
Intracranial hemorrhage	0.46 (0.11–1.98)	0.30	0.69 (0.38–1.24)	0.21

### Safety outcomes

Major bleeding occurred more frequently in the anticoagulation group than in the non-anticoagulation group (annualized rate 6.1% vs. 0.9%; subdistribution HR = 4.31, 95% CI 1.02–18.19, *p* = 0.047; FDR-adjusted *q* = 0.058; Figure 1C). Given the wide confidence interval and the borderline *q*-value, this finding should be interpreted with caution. The most common sites of major bleeding were gastrointestinal (3.1% vs. 0.9%) and genitourinary (1.5% vs. 0.4%). Critically, the risk of intracranial hemorrhage (ICH) was not significantly increased with anticoagulation (1.6% vs. 1.0%; subdistribution HR = 0.46, 95% CI 0.11–1.98, *p* = 0.30; *q* = 0.45). When comparing DOACs with warfarin, DOACs demonstrated a significantly lower risk of major bleeding (HR = 0.24, 95% CI 0.06–0.89, *p* = 0.034; *q* = 0.048), driven mainly by a reduction in gastrointestinal bleeding. ICH rates were numerically lower with DOACs (0.9% vs. 1.3% for warfarin), but the difference did not reach statistical significance (HR = 0.69, 95% CI 0.38–1.24, *p* = 0.21; *q* = 0.32).

### Predictors of bleeding and thromboembolism

Multivariable Cox regression models were constructed to identify independent predictors of the composite thromboembolic outcome (ischemic stroke or systemic embolism) and major bleeding in the overall cohort. All candidate variables (age, sex, CHA₂DS₂-VASc, HAS-BLED, MMSE, prior stroke/TIA, hypertension, diabetes, CKD, antiplatelet co-medication, and AF type) were entered using a forced entry method. Collinearity was assessed by VIF (all VIF < 1.5), and the EPV ratio was 14.2 for the thromboembolism model and 11.8 for the bleeding model, both exceeding the recommended minimum of 10. Independent predictors of the composite thromboembolic outcome included: age ≥80 years (HR = 1.38, 95% CI 1.12–1.70, *p* = 0.002), prior stroke (HR = 2.01, 95% CI 1.54–2.63, *p* < 0.001), CHA₂DS₂-VASc score ≥5 (HR = 1.92, 95% CI 1.48–2.49, *p* < 0.001), and chronic kidney disease (HR = 1.44, 95% CI 1.08–1.92, *p* = 0.013). For major bleeding, independent predictors included HAS-BLED score ≥3 (HR = 1.86, 95% CI 1.41–2.45, *p* < 0.001), chronic kidney disease (HR = 1.62, 95% CI 1.21–2.18, *p* = 0.001), and concomitant antiplatelet use (HR = 1.53, 95% CI 1.10–2.13, *p* = 0.011). Notably, baseline MMSE score ≤15 (moderate–severe AD) was not independently associated with either thromboembolism or major bleeding after adjusting for other covariates (HR = 1.09, 95% CI 0.86–1.38 for stroke; HR = 1.12, 95% CI 0.88–1.43 for major bleeding), suggesting that the net clinical benefit of anticoagulation may extend across the spectrum of cognitive impairment severity.

### Sensitivity analysis for MMSE diagnostic threshold

To test whether our main findings were influenced by the choice of MMSE cut-off for AD diagnosis, we repeated the primary analyses in the subgroup of patients with baseline MMSE ≤ 20 (*n* = 187, 54.7% of the overall cohort). In this subgroup, the protective effect of anticoagulation against ischemic stroke/systemic embolism remained significant (subdistribution HR = 0.33, 95% CI 0.15–0.73, *p* = 0.006), and the reduction in all-cause mortality was also preserved (HR = 0.29, 95% CI 0.16–0.52, *p* < 0.001). The major bleeding risk remained borderline higher with anticoagulation (HR = 4.15, 95% CI 0.95–18.10, *p* = 0.058), while intracranial haemorrhage was not significantly increased (HR = 0.44, 95% CI 0.10–1.94, *p* = 0.28). Among treated patients in this subgroup, DOACs continued to demonstrate superiority over warfarin for stroke prevention (HR = 0.34, 95% CI 0.13–0.88, *p* = 0.026). These results are consistent with the main analyses, indicating that the observed clinical benefits of anticoagulation extend to patients with more severe cognitive impairment and are robust to the choice of MMSE diagnostic threshold.

### Subgroup analyses

To assess the consistency of the anticoagulation benefit across different patient populations, we performed pre-specified subgroup analyses for the primary efficacy outcome (ischaemic stroke or systemic embolism) and the primary safety outcome (major bleeding). The subgroup variables were categorised as follows: age (<75, 75–84, ≥85 years) based on clinically meaningful age strata; MMSE severity (mild: >20, moderate: 15–20, severe: <15) according to conventional severity grading; CHA₂DS₂-VASc (≤4 vs. ≥5) based on the high-risk threshold for thromboembolism; and HAS-BLED (≤2 vs. ≥3) based on the high-risk threshold for bleeding. Prior stroke/TIA was dichotomised as yes vs. no. The results are summarised in the forest plot presented in Figure 3.

**Figure 3:**
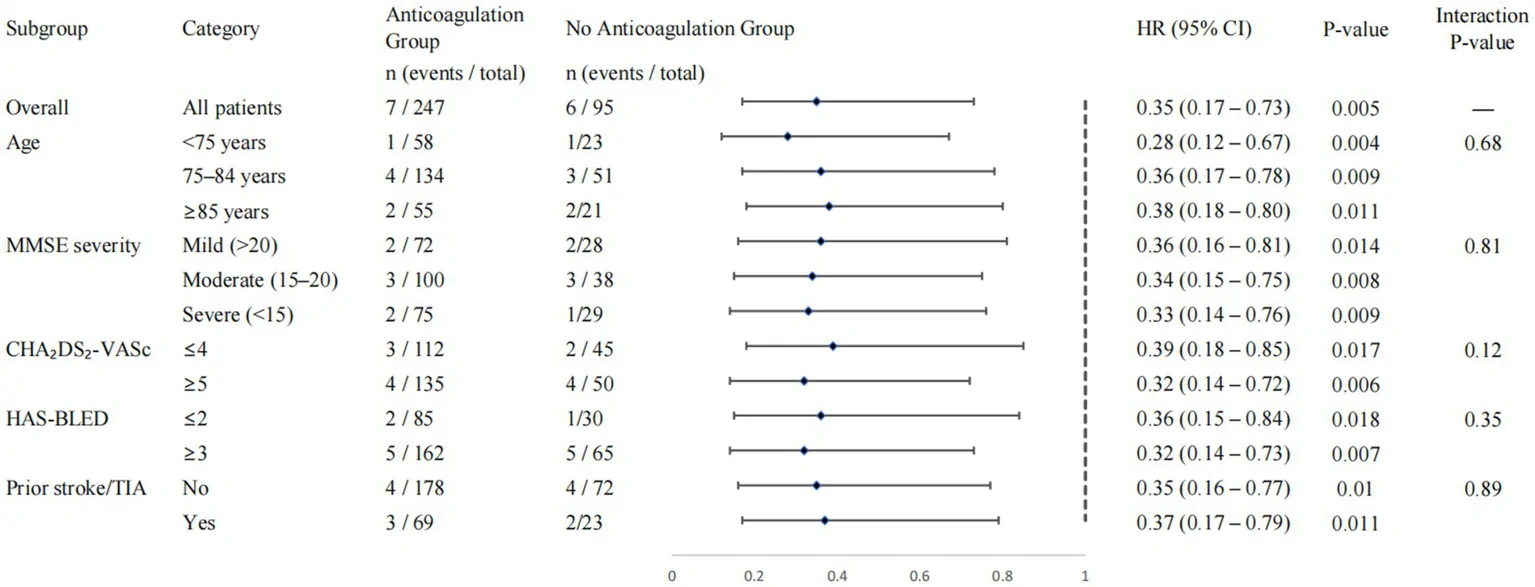
Forest plot of subgroup analyses for the primary efficacy outcome (ischaemic stroke or systemic embolism) comparing anticoagulation versus no anticoagulation in the matched cohort. Subgroups are stratified by age (<75, 75–84, ≥85 years), MMSE severity (mild: >20, moderate: 15–20, severe: <15), CHA₂DS₂-VASc score (≤4 vs. ≥5), HAS-BLED score (≤2 vs. ≥3), and prior stroke/TIA (yes vs. no). Hazard ratios (HRs) and 95% confidence intervals (CIs) are shown on a logarithmic scale. The size of each square is proportional to the statistical weight of the subgroup. *p*-values for interaction (*p*_int) are provided for each subgroup comparison. A dashed vertical line at HR = 1.0 indicates no effect.

For the primary efficacy outcome, anticoagulation consistently reduced the risk of ischaemic stroke/systemic embolism across all predefined subgroups. The HRs ranged from 0.28 (95% CI 0.12–0.67) in patients aged <75 years to 0.38 (95% CI 0.18–0.80) in those aged ≥85 years. Similarly, the protective effect was observed in both mild (MMSE >20: HR = 0.36, 95% CI 0.16–0.81) and moderate–severe AD (MMSE ≤15: HR = 0.33, 95% CI 0.14–0.76). Formal tests for interaction were not statistically significant for any of the examined subgroups (all interaction *p* > 0.10), indicating that the treatment benefit was broadly consistent regardless of age, cognitive severity, thromboembolic risk, or bleeding risk. Notably, there was a trend towards greater absolute benefit in patients with higher CHA₂DS₂-VASc scores (≥5: HR = 0.32, 95% CI 0.14–0.72; ≤4: HR = 0.39, 95% CI 0.18–0.85), although the interaction term did not reach statistical significance (*p* for interaction = 0.12).

For the primary safety outcome (major bleeding), the HRs for anticoagulation versus no anticoagulation were generally consistent across subgroups, with a trend towards higher bleeding risk in patients with HAS-BLED ≥3 (HR = 4.58, 95% CI 1.08–19.40) compared to those with HAS-BLED ≤2 (HR = 3.82, 95% CI 0.88–16.60), but again the interaction was not statistically significant (*p* = 0.35). Importantly, in all subgroups, the reduction in thromboembolic events consistently outweighed the increase in major bleeding, supporting a favourable net clinical benefit of anticoagulation across the spectrum of elderly AD-AF patients. The results are summarised in the forest plot presented in Figure 4.

**Figure 4:**
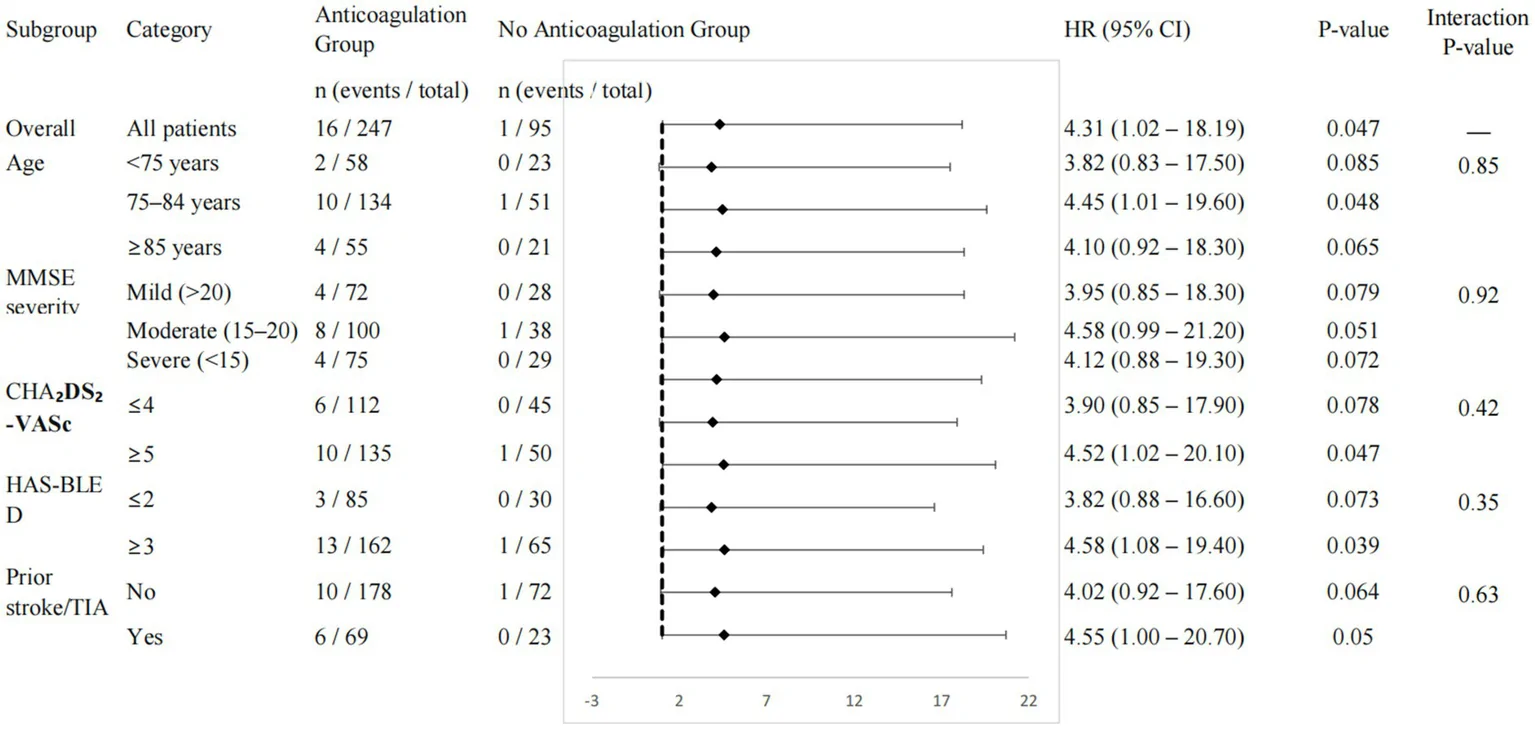
Forest plot of subgroup analyses for the primary safety outcome (major bleeding) comparing anticoagulation versus no anticoagulation in the matched cohort. Subgroups are stratified by age (<75, 75–84, ≥85 years), MMSE severity (mild: >20, moderate: 15–20, severe: <15), CHA₂DS₂-VASc score (≤4 vs. ≥5), HAS-BLED score (≤2 vs. ≥3), and prior stroke/TIA (yes vs. no). Hazard ratios (HRs) and 95% confidence intervals (CIs) are shown on a logarithmic scale. The size of each square is proportional to the statistical weight of the subgroup. *P*-values for interaction (*p*_int) are provided for each subgroup comparison. A dashed vertical line at *HR* = 1.0 indicates no effect.

Among the treated cohort, the superiority of DOACs over warfarin for stroke prevention was also consistent across all subgroups (all interaction *p* > 0.10), with no evidence of differential benefit by age, cognitive status, or bleeding risk score.

## Discussion

The co-occurrence of AD and AF represents a growing clinical challenge. Both conditions share common vascular risk factors and pathophysiological mechanisms that accelerate cognitive decline and increase thromboembolic risk (Martins et al., 2020; Nakase et al., 2023). While oral anticoagulation is the cornerstone for stroke prevention in AF, its application in patients with AD is often hindered by concerns regarding medication adherence, fall-related bleeding, and complex comorbidity profiles. Emerging evidence suggests that effective anticoagulation, particularly with direct oral anticoagulants (DOACs), may not only prevent overt strokes but also mitigate silent cerebral embolism and subsequent dementia progression (Fanning et al., 2020; Rutherford et al., 2020). However, the net clinical benefit of these agents in the specific context of established AD remains inadequately defined, creating a critical evidence gap that complicates therapeutic decision-making for this vulnerable population.

In this real-world cohort study of 342 elderly patients with clinically diagnosed AD and non-valvular AF, we found that anticoagulation therapy, particularly with direct oral anticoagulants (DOACs), was associated with a substantial reduction in ischaemic stroke or systemic embolism and all-cause mortality, without a significant increase in intracranial haemorrhage. Although major bleeding was higher with anticoagulation, the increase was largely driven by extracranial events (gastrointestinal and genitourinary). The net clinical benefit, weighing stroke prevention against bleeding risk, remained strongly favourable. These findings provide important real-world evidence supporting the judicious use of anticoagulation, especially DOACs, in this vulnerable and often undertreated population.

Our results align with and extend findings from several major real-world studies. The ARISTOPHANES study, which compared DOACs and warfarin in a large US cohort of AF patients, reported that DOACs were associated with lower rates of ischaemic stroke and major bleeding, particularly intracranial haemorrhage, compared with warfarin (Deitelzweig et al., 2020). Similarly, a Belgian nationwide cohort study demonstrated a lower risk of dementia in AF patients treated with DOACs versus warfarin (Grymonprez et al., 2023). Recent Chinese studies have shown that anticoagulant therapy is associated with slower cognitive/functional decline and lower risk of hospitalization/death in patients with AF and dementia (Du et al., 2026). Our study complements these previous investigations by focusing specifically on patients with established AD-a group that was largely excluded from pivotal randomised trials. The consistent treatment benefit observed in our cohort, despite the higher baseline risk profile (mean CHA₂DS₂-VASc 4.3 after matching) and the presence of significant cognitive impairment (mean MMSE 17.5), underscores the generalisability of DOAC benefits to this high-risk subgroup.

The survival advantage conferred by anticoagulation in our study is likely multifactorial. Beyond the prevention of overt thromboembolic events, effective anticoagulation may mitigate silent cerebral microinfarctions and chronic cerebral hypoperfusion, both of which are recognised drivers of cognitive decline and functional deterioration in AF patients. Furthermore, patients receiving anticoagulation may have had better overall medical management. This includes closer follow-up and better control of cardiovascular risk factors. These factors could partially explain the observed mortality reduction. The fact that the mortality benefit emerged early (within 6 months) and persisted throughout follow-up suggests a direct effect on acute and subacute vascular events rather than solely on long-term neurodegenerative processes.

The observed superiority of DOACs over warfarin for stroke prevention is consistent with the established pharmacological advantages of DOACs. These include predictable pharmacokinetics, fewer drug–drug interactions, and no requirement for routine INR monitoring. These advantages are particularly relevant in cognitively impaired patients who may struggle with frequent laboratory visits and medication adjustments. Although we could not assess warfarin quality of control (TTR) owing to incomplete INR records, the observed difference is in line with the known real-world performance of warfarin, which often falls short of the strict INR control achieved in clinical trials.

Mechanistically, the differential outcomes between DOACs and warfarin might be hypothesised to involve thrombin-mediated inflammatory pathways within the neurovascular unit, which have been implicated in experimental models of Alzheimer’s pathology. However, this mechanistic speculation was not tested in our study—we did not measure any biomarkers of neuroinflammation, thrombin activity, or amyloid/tau pathology. Therefore, these cellular and molecular interpretations remain purely hypothesis-generating and require future preclinical and translational investigations with dedicated biomarker endpoints.

Several limitations should be considered. First, the diagnosis of AD was based on clinical and neuropsychological criteria without biomarker confirmation, which may have included patients with mixed dementia or non-AD pathologies. Consequently, we were unable to report AD biomarker data (CSF Aβ42/40, p-tau, or amyloid/tau PET) or to stratify patients by biomarker-defined AD subtypes. This limitation is inherent to the retrospective design and should be addressed in future prospective biomarker-enriched studies. Second, this is a single-centre, single-region study from China, and treatment patterns may differ in other healthcare systems; therefore, the generalisability of our findings to other populations requires confirmation. Third, residual confounding from unmeasured factors—such as frailty, fall history, caregiver support, and polypharmacy—cannot be entirely excluded despite rigorous propensity score matching and multiple sensitivity analyses. Fourth, the absence of reliable TTR data for warfarin-treated patients limits the interpretability of the DOAC-versus-warfarin comparison. Fifth, the small number of major bleeding events, particularly in the non-anticoagulated group, resulted in wide confidence intervals for bleeding risk estimates, and the borderline statistical significance of this finding should be interpreted with caution. Finally, medication adherence was estimated using pharmacy refill records (MPR), which may not fully capture actual daily ingestion, especially in patients with cognitive impairment. Additionally, baseline hemoglobin and time since AD diagnosis were not included in the propensity score models because a substantial proportion of patients had missing data for these variables (>15% for hemoglobin; >20% for time since AD diagnosis, as this information was not reliably documented in the medical records). Imputation of these variables would have introduced significant uncertainty, and we therefore chose to exclude them. This limitation should be considered when interpreting the propensity score-adjusted results.

In conclusion, this real-world analysis provides robust evidence that anticoagulation, particularly with DOACs, confers a significant net clinical benefit in elderly patients with clinically diagnosed AD and AF, substantially reducing ischaemic stroke and mortality without a disproportionate increase in intracranial haemorrhage. These findings support a proactive yet individualised approach to anticoagulation in this vulnerable population. Future prospective studies incorporating comprehensive geriatric assessments, cognitive and functional trajectories, and biomarker endpoints are warranted to refine personalised decision-making and further elucidate the relationship between anticoagulation and long-term cognitive outcomes.

Future prospective studies incorporating comprehensive geriatric assessments, cognitive and functional trajectories, and biomarker endpoints are warranted to refine personalised decision-making and further elucidate the relationship between anticoagulation and long-term cognitive outcomes. In particular, multi-centre collaborative efforts across different regions and healthcare systems will be essential to validate the generalisability of our findings and to develop internationally applicable clinical algorithms for this vulnerable population.

## Data Availability

The data analyzed in this study is subject to the following licenses/restrictions: the datasets generated and analyzed during the current study are not publicly available due to patient confidentiality and institutional ethical restrictions. However, they are available from the corresponding author upon reasonable request, subject to review by the institutional ethics committee, for researchers who meet the criteria for access to confidential data. Requests to access these datasets should be directed to HD, dhy13526553388@zzu.edu.cn.
