# Supplementary material for: Efficacy of anticoagulation therapy in elderly patients with clinically diagnosed Alzheimer’s disease and atrial fibrillation: a clinical outcomes study
Source: Front Cell Neurosci. 2026 Sep 23;20:1874032. doi: 10.3389/fncel.2026.1874032 (PMC13642182; doi:10.3389/fncel.2026.1874032)
Supplement: Supplementary file 1 [file Table_1.docx]

| **Table S1. Covariate Balance for Anticoagulation vs. No Anticoagulation (Before Matching, After 1:1 PSM, and After IPTW)** | | | | | | | | | |
| --- | --- | --- | --- | --- | --- | --- | --- | --- | --- |
| **Covariate** | **Before Matching** | | | **After 1:1 PSM** | | | **After IPTW** | | |
|  | Anticoag (n=247) | No Anticoag (n=95) | SMD | Anticoag (n=95) | No Anticoag (n=95) | SMD | Anticoag (weighted) | No Anticoag (weighted) | SMD |
| Age, years (mean ± SD) | 78.8 ± 6.1 | 77.9 ± 6.4 | 0.14 | 78.2 ± 6.0 | 78.0 ± 6.2 | 0.03 | 78.5 ± 6.1 | 78.3 ± 6.3 | 0.03 |
| Male, n (%) | 103 (41.7) | 40 (42.1) | 0.01 | 40 (42.1) | 39 (41.1) | 0.02 | 41.50% | 42.00% | 0.01 |
| Education years (mean ± SD) | 9.4 ± 3.2 | 8.9 ± 3.5 | 0.15 | 9.1 ± 3.3 | 9.0 ± 3.4 | 0.03 | 9.3 ± 3.2 | 9.2 ± 3.3 | 0.03 |
| BMI, kg/m² (mean ± SD) | 24.8 ± 3.9 | 24.0 ± 4.1 | 0.2 | 24.4 ± 4.0 | 24.3 ± 4.0 | 0.03 | 24.6 ± 3.9 | 24.5 ± 4.0 | 0.03 |
| Hypertension, n (%) | 185 (74.9) | 67 (70.5) | 0.1 | 69 (72.6) | 68 (71.6) | 0.02 | 73.80% | 73.20% | 0.01 |
| Diabetes, n (%) | 82 (33.2) | 28 (29.5) | 0.08 | 30 (31.6) | 29 (30.5) | 0.02 | 32.50% | 32.00% | 0.01 |
| Prior stroke/TIA, n (%) | 69 (27.9) | 23 (24.2) | 0.09 | 24 (25.3) | 23 (24.2) | 0.03 | 26.50% | 25.80% | 0.02 |
| CKD, n (%) | 48 (19.4) | 14 (14.7) | 0.13 | 16 (16.8) | 15 (15.8) | 0.03 | 18.20% | 17.60% | 0.02 |
| CHA₂DS₂‑VASc (mean ± SD) | 4.6 ± 1.3 | 4.1 ± 1.5 | 0.35 | 4.3 ± 1.4 | 4.2 ± 1.4 | 0.07 | 4.5 ± 1.3 | 4.4 ± 1.4 | 0.07 |
| HAS‑BLED (mean ± SD) | 2.9 ± 0.9 | 2.5 ± 1.0 | 0.42 | 2.7 ± 0.9 | 2.6 ± 1.0 | 0.1 | 2.8 ± 0.9 | 2.7 ± 0.9 | 0.11 |
| MMSE (mean ± SD) | 17.2 ± 4.5 | 18.0 ± 4.2 | 0.18 | 17.5 ± 4.3 | 17.6 ± 4.4 | 0.02 | 17.4 ± 4.4 | 17.5 ± 4.3 | 0.02 |
| AF type (paroxysmal), n (%) | 102 (41.3) | 42 (44.2) | 0.06 | 42 (44.2) | 41 (43.2) | 0.02 | 41.80% | 42.30% | 0.01 |
| Antiplatelet co‑medication, n (%) | 31 (12.6) | 15 (15.8) | 0.09 | 13 (13.7) | 14 (14.7) | 0.03 | 12.90% | 13.40% | 0.02 |
| C‑statistic (95% CI) | 0.72 (0.68–0.76) | - | - | - | - | - | - | - | - |

Abbreviations: SMD, standardized mean difference; PSM, propensity score matching; IPTW, inverse probability of treatment weighting; CKD, chronic kidney disease; AF, atrial fibrillation.

SMD < 0.1 indicates acceptable balance.

| **Table S2. Covariate Balance for DOAC vs. Warfarin (Before Matching, After 1:1 PSM, and After IPTW)** | | | | | | | | | |
| --- | --- | --- | --- | --- | --- | --- | --- | --- | --- |
| **Covariate** | **Before Matching** | | | **After 1:1 PSM** | | | **After IPTW** | | |
|  | DOAC (n=143) | Warfarin (n=104) | SMD | DOAC (n=104) | Warfarin (n=104) | SMD | DOAC (weighted) | Warfarin (weighted) | SMD |
| Age, years (mean ± SD) | 78.4 ± 6.3 | 79.2 ± 6.0 | 0.13 | 78.9 ± 6.1 | 78.7 ± 6.2 | 0.03 | 78.6 ± 6.2 | 78.8 ± 6.1 | 0.03 |
| Male, n (%) | 60 (42.0) | 43 (41.3) | 0.01 | 44 (42.3) | 43 (41.3) | 0.02 | 41.80% | 42.00% | 0 |
| Education years (mean ± SD) | 9.6 ± 3.1 | 9.2 ± 3.4 | 0.12 | 9.4 ± 3.2 | 9.3 ± 3.3 | 0.03 | 9.5 ± 3.1 | 9.5 ± 3.2 | 0 |
| BMI, kg/m² (mean ± SD) | 24.6 ± 3.8 | 25.0 ± 4.0 | 0.1 | 24.8 ± 3.9 | 24.7 ± 3.9 | 0.03 | 24.7 ± 3.8 | 24.8 ± 3.9 | 0.03 |
| Hypertension, n (%) | 102 (71.3) | 83 (79.8) | 0.2 | 79 (76.0) | 78 (75.0) | 0.02 | 73.50% | 74.00% | 0.01 |
| Diabetes, n (%) | 46 (32.2) | 36 (34.6) | 0.05 | 34 (32.7) | 35 (33.7) | 0.02 | 33.00% | 33.20% | 0 |
| Prior stroke/TIA, n (%) | 39 (27.3) | 30 (28.8) | 0.03 | 29 (27.9) | 28 (26.9) | 0.02 | 27.50% | 27.80% | 0.01 |
| CKD, n (%) | 26 (18.2) | 22 (21.2) | 0.08 | 20 (19.2) | 19 (18.3) | 0.02 | 19.00% | 19.30% | 0.01 |
| CHA₂DS₂‑VASc (mean ± SD) | 4.5 ± 1.4 | 4.7 ± 1.2 | 0.15 | 4.6 ± 1.3 | 4.5 ± 1.3 | 0.08 | 4.5 ± 1.3 | 4.6 ± 1.2 | 0.08 |
| HAS‑BLED (mean ± SD) | 2.8 ± 0.9 | 3.0 ± 0.8 | 0.23 | 2.9 ± 0.8 | 2.8 ± 0.9 | 0.12 | 2.9 ± 0.8 | 2.9 ± 0.8 | 0 |
| MMSE (mean ± SD) | 17.5 ± 4.4 | 16.9 ± 4.6 | 0.13 | 17.2 ± 4.5 | 17.3 ± 4.4 | 0.02 | 17.4 ± 4.4 | 17.3 ± 4.5 | 0.02 |
| eGFR, mL/min/1.73m² (mean ± SD) | 72.3 ± 15.2 | 68.5 ± 16.4 | 0.24 | 70.5 ± 15.8 | 70.1 ± 16.0 | 0.03 | 71.2 ± 15.5 | 70.8 ± 15.9 | 0.03 |
| AF type (paroxysmal), n (%) | 61 (42.7) | 41 (39.4) | 0.07 | 43 (41.3) | 42 (40.4) | 0.02 | 42.00% | 41.50% | 0.01 |
| Antiplatelet co‑medication, n (%) | 18 (12.6) | 13 (12.5) | 0 | 13 (12.5) | 12 (11.5) | 0.03 | 12.40% | 12.20% | 0.01 |
| C‑statistic (95% CI) | 0.69 (0.64–0.74) | - | - | - | - | - | - | - | - |
|  |  |  |  |  |  |  |  |  |  |
| Abbreviations: DOAC, direct oral anticoagulant; eGFR, estimated glomerular filtration rate. Other abbreviations as in Table S1. | | | | | | | | | |
| SMD < 0.1 indicates acceptable balance. | | | | | | | | | |

| **Table S3. IPTW Sensitivity Analysis – Hazard Ratios for Primary Efficacy and Safety Outcomes** | | | | |
| --- | --- | --- | --- | --- |
| **Outcome** | **Anticoagulation vs. No Anticoagulation** | | **DOAC vs. Warfarin** | |
|  | HR (95% CI) | *p*‑value | HR (95% CI) | *p*‑value |
| Ischemic stroke / systemic embolism | 0.37 (0.18 – 0.75) | 0.006 | 0.38 (0.16 – 0.89) | 0.026 |
| All‑cause mortality | 0.34 (0.21 – 0.56) | <0.001 | 0.87 (0.55 – 1.37) | 0.54 |
| Major bleeding | 4.18 (1.01 – 17.30) | 0.049 | 0.26 (0.07 – 0.92) | 0.036 |
| Intracranial hemorrhage | 0.48 (0.12 – 1.92) | 0.3 | 0.71 (0.40 – 1.26) | 0.24 |
|  |  |  |  |  |
| **Abbreviations:** IPTW, inverse probability of treatment weighting; HR, hazard ratio; CI, confidence interval. | | | | |
| All models were weighted by the inverse probability of treatment and adjusted for the same covariates as in the primary PSM analyses. The IPTW results are consistent with the main matched analyses, confirming the robustness of the findings. | | | | |

| **Supplementary Table S4. STROBE Statement – Checklist of Items to Be Included in Reports of Cohort Studies** | | | |
| --- | --- | --- | --- |
| **Section & Topic** | **Item No.** | **Recommendation** | **Reported in Manuscript** |
| Title and abstract | 1 | (a) Indicate the study’s design with a commonly used term in the title or abstract | Yes (Title: "…A Clinical Outcomes Study"; Abstract: "real‑world cohort study") |
|  |  | (b) Provide in the abstract an informative and balanced summary of what was done and what was found | Yes (Abstract structured with objectives, methods, key results, conclusions) |
| Introduction |  |  |  |
| Background/rationale | 2 | Explain the scientific background and rationale for the investigation being reported | Yes (Introduction, paragraphs 1‑2) |
| Objectives | 3 | State specific objectives, including any prespecified hypotheses | Yes (Introduction, last paragraph: three objectives) |
| Methods |  |  |  |
| Study design | 4 | Present key elements of study design early in the paper | Yes (Methods, first sentence: "retrospective cohort study") |
| Setting | 5 | Describe the setting, locations, and relevant dates, including periods of recruitment, exposure, follow‑up, and data collection | Yes (Methods: single center, dates Jan 2022–Dec 2025, follow‑up to Dec 31, 2025) |
| Participants | 6 | (a) Give the eligibility criteria, and the sources and methods of selection of participants | Yes (Methods, Inclusion/Exclusion criteria) |
|  |  | (b) Describe methods of follow‑up | Yes (Methods, follow‑up at 3‑month intervals via clinic/phone/records) |
| Variables | 7 | Clearly define all outcomes, exposures, predictors, potential confounders, and effect modifiers. Give diagnostic criteria, if applicable | Yes (Outcomes defined in Methods; covariates listed in PSM and multivariable models) |
| Data sources/measurement | 8 | For each variable of interest, give sources of data and details of methods of assessment (measurement) | Yes (Data extracted from EMR; details described) |
| Bias | 9 | Describe efforts to address potential sources of bias | Yes (PSM, IPTW, blinded adjudication, competing‑risk analysis, time‑updated Cox) |
| Study size | 10 | Explain how the study size was arrived at | Yes (All eligible patients during the period; no sample size calculation for retrospective design) |
| Quantitative variables | 11 | Explain how quantitative variables were handled in the analyses. If applicable, describe which groupings were chosen and why | Yes (Continuous variables as mean±SD; categorical as n (%); groupings for MMSE, age, scores described) |
| Statistical methods | 12 | (a) Describe all statistical methods, including those used to control for confounding | Yes (PSM, IPTW, Cox, Fine‑Gray, time‑updated Cox, FDR correction) |
|  |  | (b) Describe any methods used to examine subgroups and interactions | Yes (Forest plot with interaction tests) |
|  |  | (c) Explain how missing data were addressed | Yes (Methods, missing data subsection) |
|  |  | (d) If applicable, describe analytical methods taking account of competing risks | Yes (Fine‑Gray regression) |
|  |  | (e) Describe any sensitivity analyses | Yes (IPTW, time‑updated Cox, complete‑case analysis) |
| Results |  |  |  |
| Participants | 13 | (a) Report numbers of individuals at each stage of study—e.g., numbers potentially eligible, examined for eligibility, confirmed eligible, included in the study, completing follow‑up, and analysed | Yes (Figure 1 – flow diagram) |
|  |  | (b) Give reasons for non‑participation at each stage | Yes (Figure 1) |
|  |  | (c) Consider use of a flow diagram | Yes (Figure 1) |
| Descriptive data | 14 | (a) Give characteristics of study participants (e.g., demographic, clinical, social) and information on exposures and potential confounders | Yes (Table 1 and Supplementary Tables S1–S2) |
|  |  | (b) Indicate number of participants with missing data for each variable of interest | Yes (Supplementary materials; missing data handling described) |
|  |  | (c) Summarise follow‑up time (e.g., average and total amount) | Yes (Mean and median follow‑up; at‑risk tables in Figure 2) |
| Outcome data | 15 | Report numbers of outcome events or summary measures over time | Yes (Annualised rates, HRs, 95% CIs in Table 2 and text) |
| Main results | 16 | (a) Give unadjusted estimates and, if applicable, confounder‑adjusted estimates and their precision (e.g., 95% CI). Make clear which confounders were adjusted for and why they were included | Yes (Adjusted HRs from PSM/IPWT models; confounders listed) |
|  |  | (b) Report category boundaries when continuous variables were categorised | Yes (e.g., age groups, MMSE strata, score thresholds) |
|  |  | (c) If relevant, consider translating estimates of relative risk into absolute risk for a meaningful time period | Yes (Absolute risk reduction, NNT) |
| Other analyses | 17 | Report other analyses done—e.g., analyses of subgroups and interactions, and sensitivity analyses | Yes (Sub group forest plot, IPTW, time‑updated Cox, complete‑case analysis) |
| Discussion |  |  |  |
| Key results | 18 | Summarise key results with reference to study objectives | Yes (Discussion, first paragraph) |
| Limitations | 19 | Discuss limitations of the study, taking into account sources of potential bias or imprecision. Discuss both direction and magnitude of any potential bias | Yes (Limitations paragraph) |
| Interpretation | 20 | Give a cautious overall interpretation of results considering objectives, limitations, multiplicity of analyses, results from similar studies, and other relevant evidence | Yes (Discussion, throughout) |
| Generalisability | 21 | Discuss the generalisability (external validity) of the study results | Yes (Limitations section: single‑centre, China) |
| Other information |  |  |  |
| Funding | 22 | Give the source of funding and the role of the funders for the present study and, if applicable, for the original study on which the present article is based | Yes (Funding statement in Acknowledgements – to be added if applicable) |

| Supplementary Table S5. Sensitivity Analysis Using Time‑Updated Cox Regression – Hazard Ratios for Primary Efficacy and Safety Outcomes | | | | |
| --- | --- | --- | --- | --- |
| **Outcome** | **Anticoagulation vs. No Anticoagulation** | | **DOAC vs. Warfarin** | |
|  | HR (95% CI) | *p*‑value | HR (95% CI) | *p*‑value |
| Ischemic stroke / systemic embolism | 0.37 (0.18 – 0.76) | 0.007 | 0.39 (0.17 – 0.90) | 0.028 |
| All‑cause mortality | 0.33 (0.20 – 0.55) | <0.001 | 0.88 (0.54 – 1.42) | 0.59 |
| Major bleeding | 4.22 (1.03 – 17.30) | 0.045 | 0.25 (0.07 – 0.93) | 0.038 |
| Intracranial hemorrhage | 0.47 (0.12 – 1.85) | 0.28 | 0.72 (0.41 – 1.28) | 0.26 |
| Abbreviations: HR, hazard ratio; CI, confidence interval. | | | | |
| Method: Anticoagulation exposure was treated as a time‑dependent covariate in Cox regression models. Patients could change exposure status (start, switch, or discontinue) during follow‑up; the model updated their exposure at each event time. All models were adjusted for the same baseline covariates as in the primary PSM analyses (age, sex, education, BMI, hypertension, diabetes, prior stroke/TIA, CKD, CHA₂DS₂‑VASc, HAS‑BLED, MMSE, AF type, antiplatelet use). The results are consistent with the main Fine‑Gray competing‑risk analyses, confirming that the observed protective effects of anticoagulation (particularly DOACs) are robust to changes in treatment status over time. | | | | |
